# Detection of Asymptomatic Sickle Cell Hemoglobin Carriers and Fetal Hemoglobin Regulating Genetic Variants in African Descendants from Oaxaca, Mexico

**DOI:** 10.1155/2024/4940760

**Published:** 2024-04-29

**Authors:** María De Los Ángeles Romero-Tlalolini, Sergio Roberto Aguilar-Ruiz, Rafael Baltiérrez-Hoyos, Jaime Vargas-Arzola, Luis Alberto Hernández-Osorio, Verónica Rocío Vásquez-Garzón, Héctor Ulises Bernardino-Hernández, Honorio Torres-Aguilar

**Affiliations:** ^1^CONACYT-Medicine and Surgery Faculty, Universidad Autónoma “Benito Juárez” de Oaxaca, Oaxaca, Mexico; ^2^Chemical Sciences Faculty, Universidad Autónoma “Benito Juárez” de Oaxaca, Oaxaca, Mexico

## Abstract

Sickle cell anemia has been classified as a noninfectious neglected tropical disease and, although not exclusively, affects African descendants more frequently. This study aimed to detect asymptomatic sickle cell hemoglobin carriers (HbAS) in marginalized and vulnerable populations during a public health screening in African descendants from Oaxaca, Mexico, and to validate an amplification refractory mutation system (ARMS)-PCR methodology to detect fetal-hemoglobin (HbF)-regulating genetic variants in BCL11A toward affordable routine association of single nucleotide variants (SNVs) with HbF concentrations. To this aim, hemoglobin variants were detected by acidic citrate agar and alkaline cellulose acetate electrophoreses. SNVs in the hemoglobin subunit beta gene (HBB) were identified by the *β*-globin mutation detection assay (*β*-GMDA) and ARMS-PCR, respectively, and validated by Sanger sequencing. The association between genotypes and HbF concentrations was evaluated using Spearman's correlation coefficient. The results obtained during a directed screening in 140 self-identified African descendants revealed 42 HbS-carriers (30%), of which 39 showed normal total hemoglobin concentrations (92.8%), only 3 presented anemia (7.2%), and 9 showed quantifiable HbF concentration (21.4%). As validated by Sanger sequencing, the designed ARMS-PCR efficiently detected homozygous and heterozygous variants in BCL11A. In a cohort of 42 heterozygous (HbAS) and 27 healthy (HbAA) individuals from the same population, only one SNV (rs766432) showed statistically significant association with increasing HbF concentration, and two new unrelated homozygous silent variants were identified. This study reveals the need to raise coverage of HbS screening in vulnerable populations and shows a feasible low-cost ARMS-PCR methodology to determine the presence of SNVs in quantitative trait loci affecting HbF.

## 1. Introduction

Because of its clinical and epidemiologic features, sickle cell anemia (SCA) has been classified as a noninfectious neglected tropical disease that constitutes a chronic and debilitating condition that disproportionately affects populations living in extreme poverty [1, 2]. SCA is an autosomal recessive disorder mainly affecting individuals of African descent caused by nucleotide change at codon 7 in the HBB (Hemoglobin subunit beta) gene (HBB.:c.20A > T, p.Glu6Val). The physiopathology of this condition is characterized by the formation and polymerization of sickle hemoglobin (HbS), giving rise to rigid sickle-shaped erythrocytes. Such cell deformation may originate unpredictable vaso-occlusive crises with heterogeneous clinical manifestations, including acute pain, chronic inflammation, and hemolytic anemia, until early death [3].

A heterozygote carrier state (HbAS) is not sickle cell disease. Variability in severity arises when heterozygous forms of the HBB gene occur, such as HbSC and HbS/beta-thalassemia diseases. Additionally, the clinical variability is explained in part by environmental factors but also by genetic features such as heterozygous (HbAS) or homozygous (HbSS) variants and by a higher fetal hemoglobin (HbF) concentration, which has been recognized as a favorable modifier of severity in SCA due to its ability for inhibiting the polymerization and reducing the mean corpuscular concentration of HbS. However, HbF levels may considerably differ due to genetic determinants such as single nucleotide variants (SNVs) [4, 5].

Three significant quantitative trait loci (QTLs), designated BCL11A, HMIP-2, and XmnI-HBG, have been associated with fetal hemoglobin concentrations (HbF QTLs). SNVs in HbF QTLs have favorable quantitative effects on HbF expression, explaining 20–50% of HbF variation in SCA patients and being relevant for their treatment, follow-up, and prognosis [6, 7].

Compared to other currently described HbF QTLs, BCL11A (B-cell CLL/lymphoma 11A), located on chromosome 2p16, has shown more significant and reproducible associations and has been validated as a *γ*-globin gene silencer (HbF contains two *α* and two *γ* globins). When this study started, regardless of the regulatory mechanism, genetic observations supported that BCL11A expression was influenced by six main SNVs, highlighting these variants in intron II (rs1427407, rs7599488, rs766432, rs11886868, rs4671393, rs7557939). These SNVs had been associated with HbF repression in late-stage erythroid cells, where decreased BCL11A expression correlates with increased HbF concentrations, as well as clinical and hematological parameters, reflecting a decrease in clinical severity of sickle cell disease [8–10].

Although no sickle cell patients were found, this study detected asymptomatic HbAS-carrier individuals during public health screening in individuals of African descent. An Amplification Refractory Mutation System (ARMS) PCR was designed and validated by Sanger sequencing to detect the presence of the six major non-coding SNVs in the HbF QTLs, BCL11A. Its functionality was demonstrated by revealing heterozygous and homozygous individuals for each SNV and a correlational analysis with their HbF concentrations.

## 2. Materials and Methods

### 2.1. Individuals and Blood Tests

Blood samples (5 mL per person) were collected during a non-probability convenience sampling of individuals who voluntarily accepted to participate in HbS detection and self-identified as afro-descendants during a public health screening in Santa María Cortijo and Rio Grande, Oaxaca, Mexico communities. All individuals were analyzed by routine blood tests to evaluate their general health status, including a complete blood count (hematology analyzer Advia 60) and serum iron test (Biosystems). Samples were obtained before authorization under informed consent in adults or guardian approval in minors. The project was approved by the research and ethical committees of the Biochemical Sciences Faculty of the Autonomous University “Benito Juárez” of Oaxaca (number: FCQ/CEI/001/2016).

### 2.2. Detection and Quantification of Hemoglobin Variants

The presence of hemoglobin variants was identified by manual electrophoreses, including the acidic citrate agar (pH = 6.0, 40 V, 4°C, 200 min) and the alkaline cellulose acetate (pH = 8.6, 250 V, 25°C, 60 min) methods, including the hemoglobins control AFSA2 SCE127E INTERLAB®. The densitometric analysis for hemoglobin quantification was performed using Image Studio Lite software. Blood samples with HbS were screened using the sodium metabisulfite test to evaluate sickle cell-shaped erythrocyte induction under brightfield microscopy at 40X.

### 2.3. DNA Samples

Genomic DNA was extracted from total leukocytes from individuals by the phenol-chloroform method and the HeLa cell line, as a positive internal control for the presence of the SNVs because this cell line genome has been demonstrated as providing a nucleotide-resolution view of a significant number of mutations [11, 12]. DNA purity, integrity, and concentration were quantified by spectrophotometric analysis, and samples were aliquoted and stored at 4°C until use.

### 2.4. Amplification and Sequencing of the HBB Gene

The HBB gene was amplified and sequenced from twelve nonrelated individuals with HbS and one HbAA subject as control. Heritable large mutations in the HBB gene are rare, but the 619 bp deletion (Δ619 bp) encompassing the exon 3 has been reported with high prevalence [13]. Hence, to detect the pathogenic sickle cell anemia-associated variant rs334 and other SNVs in the HBB gene, the *β*-globin mutation detection assay system (*β*-GMDA) proposed by Chan OT et al. [14] was performed to amplify and sequence the HBB gene. Briefly, the variants were evaluated in three PCR reactions using the primers described in Table 1: Initially, a PCR-I (primers A and B) including exon 1, intervening sequences-I (IVS-I), and exon 2 with a 1457-bp size product. PCR-I was followed by the PCR-II (primers C and D) designed to amplify exon 3 and a part of IVS-II with a 1213-bp size product or 594-bp if the 619-bp fragment is deleted. When PCR-I did not amplify because the Δ619 bp was present, a secondary reaction called PCR-III (primers A and E) was employed to bypass the 619-bp deletion using an antisense primer located before the deleted region; this reaction amplified a 695-bp size product.

### 2.5. ARMS-PCR and Validation by Sanger Sequencing Method

The sequence corresponding to the gene BCL11A was obtained from the NCBI database (https://www.ncbi.nlm.nih.gov/) in FASTA format (RefSeq NG_011968.1). The SNVs of interest (rs1427407, rs7599488, rs766432, rs11886868, rs4671393, rs7557939) were visualized in the variation viewer of the NCBI (https://www.ncbi.nlm.nih.gov/variation/view) and then located along with their flanking sequences in the sequence obtained in FASTA format. The allelic discrimination of each SNV was performed by ARMS-PCR, for which different primers were designed containing the nucleotide corresponding to the SNV to evaluate, at the 3′ end in sense or antisense primer, according to their optimal physicochemical properties.  Table 2 shows the designed primers for SNV evaluation.

The polymorphic region within IVS-II of BCL11A is 3.7 kb long. Therefore, three PCR products covered the entire area to optimize the sequencing process. The fragments obtained were sequenced by Macrogen Inc ® with the primers listed in Table 3. The generated sequences were analyzed using multiple sequence alignment by Florence Corpet and by the Ape Sec bioinformatics software for SNV identification.

### 2.6. Statistical Analysis

The correlation between the categorical qualitative variable (genotype defined as the presence or the absence of each SNV) and the quantitative variable (HbF concentration) was evaluated based on Spearman's correlation coefficient (Rho). Hence, a positive correlation close to +1 signifies that the presence of a specific SNV tends to increase the HbF concentration. Statistical analysis was performed on SPSS 21.0 software (SPSS Inc.; Chicago, IL, USA).

## 3. Results

### 3.1. The Screening of Hemoglobin Variants Revealed the Presence of Asymptomatic HbS Carriers

Santa María Cortijo and Rio Grande are villages located 104 and 37 meters above sea level, respectively, on the Oaxaca state coast, Mexico. Both communities are described as having a high density of individuals of African descent by the National Institute of Statistics and Geography of Mexico [15]. The proximity and similarity of both villages prompted us to calculate average hemoglobin values ± SD for healthy individuals as a common point of reference, giving ranges of 11.0–14.3 g/dL for women and 12.2–15.5 g/dL for men.

**Figure 1:**
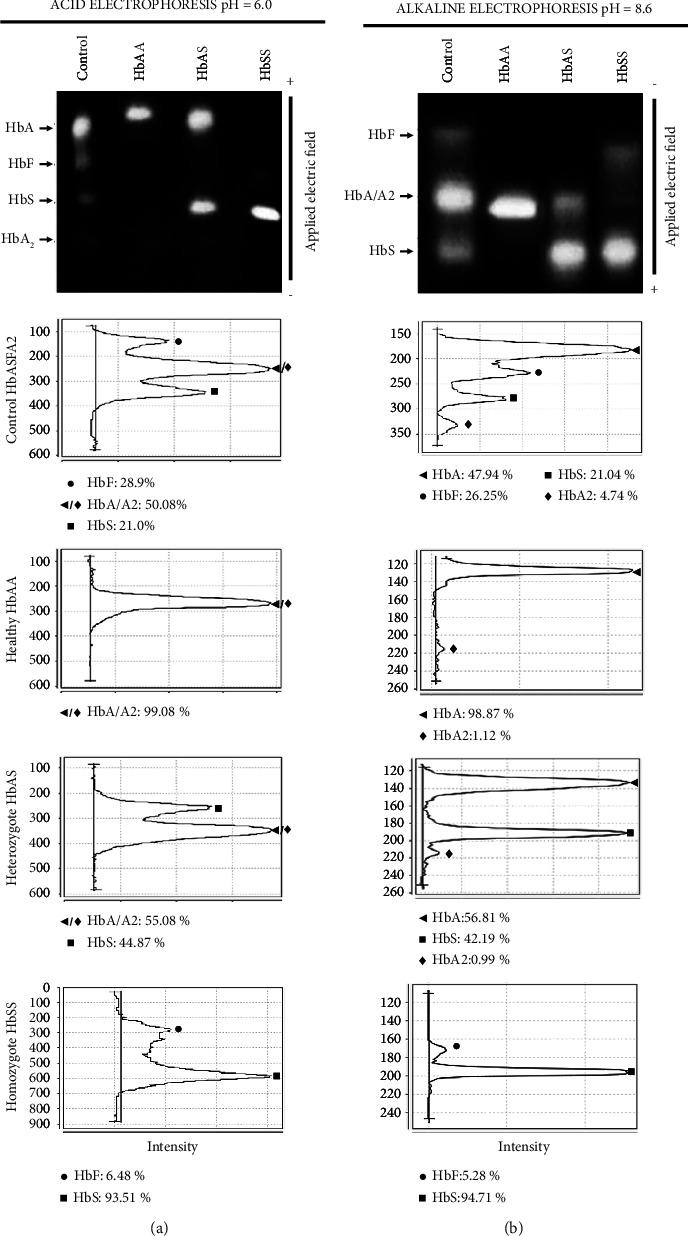
Detection and quantification of hemoglobin variants. Blood samples were analyzed by manual electrophoreses: (a) acidic citrate agar and (b) alkaline cellulose acetate as described in Materials and Methods. Images show representative results of electrophoreses, and densitograms show the quantification of the detected hemoglobins in healthy (HbAA), heterozygous (HbAS), and homozygous (HbSS) individuals in comparison to the control containing A, A2, F and S hemoglobins. ◀ = HbA, ♦ = HbA2, ◀/♦ = HbA/A2, ● = HbF, ■ = HbS.

140 self-identified afro-descendants volunteered in HbS detection; those with low total hemoglobin concentrations and normal serum iron levels were elected to assess the presence of structural hemoglobin variants by acidic and alkaline electrophoreses. The screening evidenced 42 HbS carriers (30% of the sampled population), of which 4 and 6 subjects were from two families and 32 were unrelated. The existence of HbS in all those samples was corroborated by the sodium metabisulfite test, as evidenced by the induction of sickle cell-shaped erythrocytes.  Figure 1 illustrates representative results of acidic citrate agar and alkaline cellulose acetate electrophoreses, as well as the densitometric analysis for the hemoglobins' quantification.

Interestingly, of the 140 participants, three HbS-negative individuals showed quantifiable HbF concentrations above 2.0%, as shown in Table 4, together with measurements for the nine HbAS samples with the highest HbF concentrations.

### 3.2. Validation of (HBB:c.20A > T, p.Glu6Val) Substitution and Identification of New Silent Mutations

**Figure 2:**
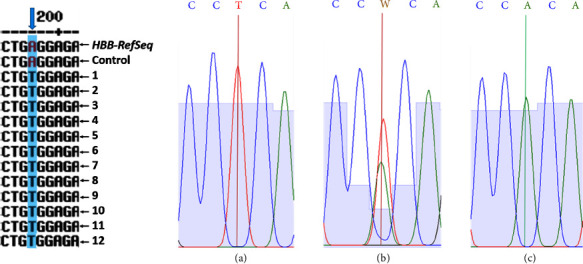
Validation of (HBB:c.20A > T, p.Glu6Val) substitution. Sequences alignment of products using primers A-E corresponding to Exon 1, IVS-I, and Exon 2. The sequences for the HBB gene (NCBI RefSeq NG_059281.1), one control of a healthy individual, and the twelve HbAS individuals are depicted in the left panel. The vertical line in the electropherograms indicates the [A > T] substitution analyzed and corresponds to (a) Control of a healthy individual (HbAA). (b) Heterozygous Individual (HbAS) and (c) Control of homozygous individual (HbSS). W = A, T.

Aiming to evaluate variations in the HBB gene from HbAS individuals, twelve samples were selected by fulfilling the following selection criteria: HbS presence by electrophoresis; positive for sickle cell induction; and DNA quantification ≥200 ng/µl with acceptable quality and integrity. Twelve HbS-carrier individuals from both municipalities and only one member per family were selected. One healthy HbAA subject from the same population was also included as a control. As shown in Figure 2, the twelve chosen samples presented the heterozygous (HBB:c.20A > T, p.Glu6Val) variation (rs334, RefSeq NM_000518.5) compared to the HBB NCBI database (RefSeq NG_059281.1) and the control sequences. The results showed that the selected individuals were not carriers of the 619-bp deletion since, in all of them, the amplification of the fragment with primers A-B was obtained, and the BLAST analysis confirmed the sequences' concordance and identities.

**Figure 3:**
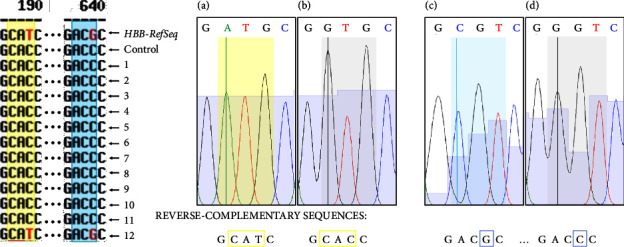
Identification of new silent variants in the analyzed population. Sequences alignment showing the sequence for HBB gen (NCBI database RefSeq NG_059281.1), one control of a healthy individual, and the twelve HbA-HbS individuals. Representative electropherograms of the CA**U**/CA**C** and AC**C**/AC**G** variations. (b) and (d) unaffected individuals. (a) and (c) Affected individuals. Variations are marked with a vertical line showing the substituted nucleotide. The RV primer was used for sequencing, so the reverse complementary sequence obtained in the electropherogram was used in the alignment.

In addition, two unrelated homozygous variants were identified in the analyzed population since eleven of the HbS-analyzed individuals and the healthy individual (control) from the same inhabitants also presented both variants (Figure 3). These variants were in codons 3(CAU/CAC), and this is a silent variant since, despite the nucleotide substitution, this produces no changes in the amino acid translation. The CAU sequence in codon 3 codifies histidine, corresponding to the original sequence reported in the NCBI database for the HBB gene. The other variant occurs in the second intron and corresponds to a non-coding region. Genetic searching in public datasets (https://www.internationalgenome.org) revealed no registered association between these mutations and other ancestral populations such as Amerindian, African, or Hispanic/Latinos.

### 3.3. ARMS-PCR Efficiently Detects Homozygous and Heterozygous SNVs in BCL11A

**Figure 4:**
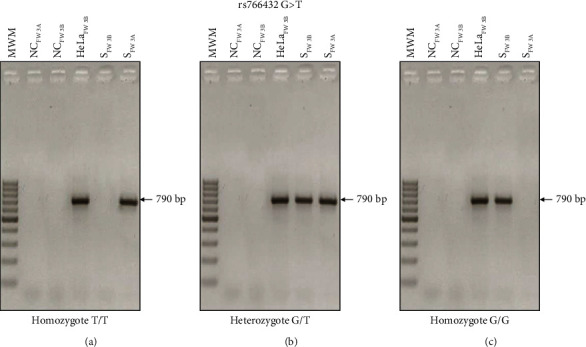
Representative results of the rs766432 SNV by ARMS-PCR. Images show the three possible outcomes when evaluating the rs766432 SNV genotype. Similar results were obtained when assessing the other SNVs. (a) Homozygous individual for alleles with the highest frequency T/T. (b) G/T heterozygous individual and (c) Homozygous individual for alleles with the lower frequency GG. MWM: Molecular weight marker; NC: Negative control; HeLa: positive internal control of HeLa DNA; S: sample, FW 3A and FW 3B: Allele-specific primers forward; RV3: reverse primer with an SNV-free binding site.

The SNVs in BCL11A (rs1427407, rs7599488, rs766432, rs11886868, rs4671393, rs7557939) were evaluated in 42 heterozygous (HbAS) and 27 healthy (HbAA) individuals by ARMS-PCR as described in Materials and Methods. The presence of heterozygous or homozygous individuals for alleles with the highest or the lower frequency for each SNV was evaluated, as illustrated for the SNV rs766432G/T in Figure 4.

Panels (A), (B), and (C) show no amplification in the negative control using any pair of primers. HeLa DNA revealed an actual amplification when using primers FW 3B-RV 3, demonstrating the efficiency of this method in detecting the presence of this SNV. Panel (A) shows amplification in HbS-carrier DNA only when using primers FW 3A-RV3, revealing a result of a homozygous individual for alleles T/T. On the other hand, panel (B) illustrates a result of a heterozygous individual G/T due to the amplification with both pairs of primers FW 3A-RV3 and FW 3B-RV3. Finally, in Panel (C), because of the amplification only with the primers FW 3B-RV3, it exemplifies a result of a homozygous individual for alleles GG.

**Figure 5:**
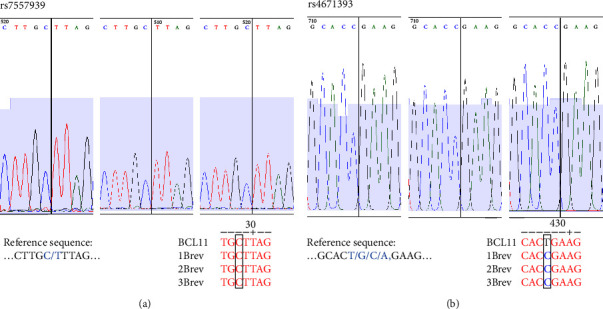
Representative results of the validation of the ARMS-PCR. (a) Sanger sequencing for validation of rs75573933 of the three samples homozygous for the allele with the highest frequency; (b) validation of rs4671393 of the three samples homozygous for the alternative allele with lower frequency.

ARMS-PCR results were validated by Sanger sequencing of three DNA samples from individuals presenting more homozygous and heterozygous variants associated with HbF concentrations (Figure 5). Panel (A) shows the identification of the homozygous nucleotide corresponding to the variants with the highest frequency of the polymorphism rs7557939; panel (B) shows the homozygous nucleotide corresponding to one of the variants with a low frequency of the polymorphism rs4671393. All the other variants were also validated by Sanger sequencing of three samples.

### 3.4. SNV (rs766432) in BCL11A Is Associated with Fetal Hemoglobin Concentration in Asymptomatic Carriers (HbAS) Individuals of African Descent

**Figure 6:**
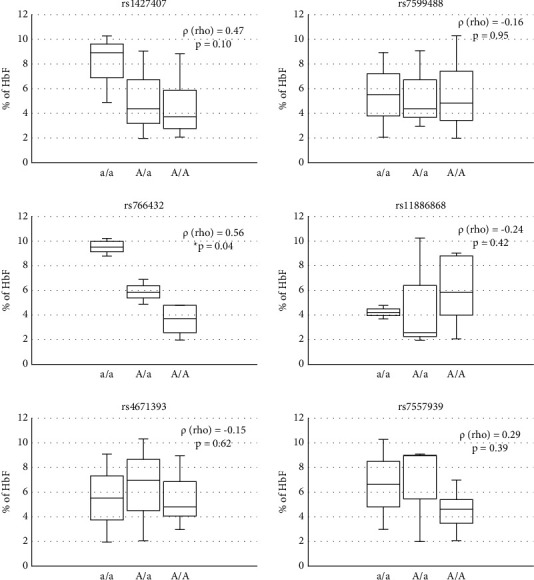
The rs766432 SNV in BCL11A is associated with HbF concentration in asymptomatic carriers (HbAS) individuals of African descent. Graphics show the correlation between genotype and HbF concentrations in nine HbAS and three HbAA individuals of African descent. The lines of the boxes represent the lower quartile, the median, and the upper quartile, respectively. a/a = Homozygous, individual for alleles with the lower frequency. A/a = heterozygous individual. A/A = Homozygous individual for alleles with the highest frequency. The correlation between the categorical qualitative variable (genotype) and a quantitative variable (HbF concentration) was evaluated by Spearman's correlation = *ρ* (rho). ^*∗*^*p* = statistically significant difference.

Because not all HbS-carrying individuals had shown detectable HbF concentrations, and some healthy individuals had shown quantifiable HbF concentrations, the statistical analysis was performed by evaluating correlations between the observed genotype, homozygote for alleles with the lower frequency (a/a), heterozygote (A/a), or homozygote for alleles with the highest frequency (A/A) of each SNV, and the HbF concentration in nine HbAS (carriers) and three HbAA (healthy) individuals who had presented quantifiable HbF by electrophoresis (Figure 6). Only the rs766432 SNV was statistically significantly associated with HbF concentration in the evaluated population. This correlation was ascendant positive when the lower frequency allele was present, so it was significant in a homozygous state and more substantial in a heterozygous state.

## 4. Discussion

Sickle cell anemia (SCA) is a common monogenic disorder that, although not exclusively, affects individuals of African descent more frequently [3]. In particular, the Oaxaca state coast of Mexico displays a high presence of this population due to migration since the beginning of the seventeenth century [15]. The directed sampling of 140 self-identified individuals of African descent from this region revealed 42 (30%) asymptomatic HbS carriers with no hematologic abnormalities or anemia as widely described [16].

These results indicate that basic low-cost techniques such as electrophoresis and sickle cell-shaped erythrocyte induction remain functional as screening methodologies and expose the need to increase coverage for future screenings in vulnerable populations to provide them with genetic counseling and efficient treatments under stressful situations.

Since this study sampling was conducted using a non-probability convenience method, it might have affected the sample's representativeness since calculating probabilistic measures such as the frequency of asymptomatic (HbAS) carriers or the presence of specific SNVs in individuals of African descent from the Oaxaca state coast might be uncertain for epidemiological studies or clinical applications. Nevertheless, because the studied populations are marginalized, the sample size represented 14.42% of the inhabitants during the study period; therefore, the high percentage of HbS carriers in the sample (30%) indicates a likely high prevalence of the variants in these communities and establish an outlook toward further epidemiological studies. Likewise, due to the research detected undiagnosed asymptomatic HbS carriers lacking clinical data, discussing these results in contrast to evidence shown by other authors in diagnosed SCA patients looking for clinical correlations is not feasible. A further evaluation, including more SNV in other HbF QTL, toward detecting prognostic markers in diagnosed SCA patients would require a genetically characterized newborn cohort with clinical prospective follow-up over time.

Nevertheless, interestingly, the amplification and sequencing of the *β*-globin gene in the twelve HbAS and one HbAA selected individuals revealed likely new silent homozygous variants in this population. The likelihood of generating homozygous mutations is very low because the two HBB alleles have different origins from each descent; hence, their presence might be because of the high rate of endogamy in this population [15], whose practice would increase the transference of this and other variants, including those causing other hemoglobinopathies. In this regard, populations with endogamous unions have been reported with high rates and common inherited blood disorders [17]. Therefore, premarital screenings have been applied to detect hemoglobinopathies carriers and genetic counseling programs, whose results have successfully reduced the prevalence of these inherited blood disorders [18]. Additionally, evaluating the probable association of these newly reported variants with the presence of beta-thalassemia deletions and other inherited blood disorders would reduce the frequency of these diseases and provide directed treatments for diagnosed patients. However, given the sample size and because the research was performed only in two populations from Oaxaca's coast, more extensive and statistically representative sampling is needed to reach such conclusions.

Several techniques, such as restriction enzymes, TaqMan probes, or direct sequencing, have been used to determine the SNVs in HbF QTLs analyzed in this study and other SNVs regarding ethnic differences in the haplotypes of interest [8, 19, 20]. Nevertheless, given the need to detect potential HbS carriers due to the symptoms presented by some residents of the evaluated regions and because of the equipment constraints and limited funding faced during the research, this study included manual electrophoreses for detecting hemoglobin variants, and it developed a low-cost ARMS-PCR-based strategy for these same goals. Likewise, aiming to increase the reliability of the results, these were validated by Sanger sequencing, whose analysis showed the feasibility of the designed ARMS-PCR methodology to apply it for diagnosis, personalized treatment, and prognosis in SCA patients. In addition, because of the ascendant significant genotype-dependent association of the SNV (rs766432) in BCL11A with increasing HbF concentration, this SNV might be further evaluated for clinical associations. Likewise, it would be necessary to design additional ARMS-PCR to evaluate more SNVs and extend the panels for different populations. In this regard, a recent study of thirty-nine diagnosed SCA individuals from South Mexico showed significant HbF elevation with a different SNV in BCL11A (rs11886868) and only in the homozygous HbSS patients [21]. Likewise, other investigations worldwide have reported the rs11886868 variant as representative of the BCL11A-QTL [7, 22]. Such discrepant results might be attributable to multiple variables such as ethnicity and medical history, but mainly to the limited number of samples and the low frequency of the different genotypes, as shown by Ghamrawy et al. in 2020 in an Egyptian SCA population [10].

## 5. Limitations of the Study

The directed non-probabilistic convenience method used for sampling limits the calculation of the frequency of asymptomatic HbS carriers or specific SNVs in the studied population. The lack of further clinical data (besides the HbF concentration) to evaluate potential correlations reported by other authors is not feasible.

## 6. Future Research Directions and Implications for Clinical Practice

This study highlights the need to raise coverage of HbS screening in vulnerable populations to provide them with genetic counseling. It is necessary to design additional ARMS-PCR to evaluate more SNVs in additional HbF QTL associated with each specific population. Conducting regional multicenter studies to search for clinical correlations in asymptomatic and diagnosed patients would provide the prognosis value on the HbF expression for the presence or absence of each specific SNV directed toward the SCA patients' treatment, follow-up, and prognosis.

## 7. Conclusions

This research revealed the existence of asymptomatic HbS-carriers in African descendants from vulnerable populations at the coast of Oaxaca, Mexico. The designed ARMS-PCR efficiently detected homozygous and heterozygous variants in BCL11A; hence, this methodological strategy might be applied to detect more SNVs in other HbF QTLs. The SNV (rs766432) showed a statistically significant association with increasing HbF concentration, yet its likely clinical relevance needs further research in patients with sickle cell anemia.

## Figures and Tables

**Table 1:** HBB-specific primers [14].

Primer	Sequence 5′ ⟶ 3′
A (forward)	ACGGCTGTCATCACTTAGAC
B (reverse)	AAGAGGTATGAACATGATTAGC
C (forward)	GTGTACACATATTGACCAAATC
D (reverse)	CAGATTCCGGGTCACTGTG
E (reverse)	CTCCCCTTCCTATGACATGA

**Table 2:** Primers' sequences for detection of SNVs-HbF in BCL11A through ARMS-PCR.

SNV ID	Primer	Sequence 5′ ⟶ 3′
rs1427407	FW 1	AGTTAGGACTTCCTTTTACTGTACT
	**RV 1A**	TAACCTTCTTAGCACCCACAAACA**T**
	**RV 1B**	TAACCTTCTTAGCACCCACAAACA**C**

rs7599488	FW 2	AACACGGAGTGATGATGCCTAGGGT
	**RV 2A**	CAAAGAAGTTAGTCTCAGCCACCTG**A**
	**RV 2B**	CAAAGAAGTTAGTCTCAGCCACCTG**G**

rs766432	**FW 3A**	TGAATGACTTTTGTTGTATGTAAA**G**
	**FW 3B**	TGAATGACTTTTGTTGTATGTAAA**T**
	RV 3	ATACTGATGAATAAGACTGAGTT

rs11886868	FW 4	GAGGTTTCTATTCGGAATAGGA
	**RV 4A**	ATCGTCTTTTGTGTTTAATTTCTT**C**
	**RV 4B**	ATCGTCTTTTGTGTTTAATTTCTT**A**

rs4671393	FW 5	GGTTTCTTAAGTAATGTAGGTG
	**RV 5A**	GCTGTGGACAGCAAAGCTTC**A**
	**RV 5B**	GCTGTGGACAGCAAAGCTTC**C**

rs7557939	**FW 6A**	CAGCATCACCCTCTCTCACTCTTG**C**
	**FW 6B**	CAGCATCACCCTCTCTCACTCTTG**T**
	RV 6	GTTGACCTCCCCCATTAGCAGCATG

Three primers were designed to identify the presence of each SNV: one primer with an SNV-free binding site paired with each primer to differentiate the base in the SNV. Additionally, one primer containing at the 3′ end one possible nucleotide of the SNV was evaluated, so the bold values in the primer column represent the alternative primers used to detect the SNVs, and the bold letters in the Sequence 5′ ⟶ 3′ column indicate the alternative nucleotides.

**Table 3:** Primers for Sanger sequencing of BCL11A.

Primer	Sequence 5′ ⟶ 3′
BCL11A RV7	GTTGACCTCCCCCATTAGCAGCATG
BCL11A RV8	ATCGTCTTTTGTGTTTAATTTCTT
BCL11A FW7	AACACGGAGTGATGATGCCTAGGGT

**Table 4:** Percentages of hemoglobin variants identified in asymptomatic carriers (HbAS) and healthy individuals (HbAA).

Number	HbA%	HbA_2_%	HbF %	HbS %	Gender	Age
*Asymptomatic carriers (HbAS)*						
1	50.29	1.72	9.05	38.94	M	3
2	49	0.99	8.92	41.09	F	58
3	48.15	0.95	8.82	42.08	F	27
4	43.08	1.92	6.93	48.07	M	3
5	49.48	1.02	4.87	44.63	F	10
6	49.18	1.95	4.79	44.08	M	4
7	47.61	0.95	4.37	47.07	M	8
8	49.36	1.07	3.71	45.86	F	61
9	53.59	1.09	2.96	42.36	F	3

*Healthy individuals (HbAA)*						
1	95.32	2.11	2.57	0	F	25
2	95.57	2.36	2.07	0	F	4
3	95.86	2.18	2.96	0	F	47

Percentages were quantified by densitometric analysis using Image Studio Lite software based on alkaline cellulose acetate electrophoresis results. Data are displayed in descending order based on fetal hemoglobin concentration. M = Male, F = Female.

## Data Availability

The data used to support the findings of this study are included in the article. Additional databases are available for further research upon request from the corresponding author.
